# Research on re-searching: interrupted foraging is not disrupted foraging

**DOI:** 10.1186/s41235-024-00556-8

**Published:** 2024-05-15

**Authors:** Injae Hong, Jeremy M. Wolfe

**Affiliations:** 1 Visual Attention Lab, Brigham and Women’s Hospital, Boston, MA 02135 USA; 2 Harvard Medical School, Boston, USA; 3 Yonsei University, Seoul, South Korea

**Keywords:** Foraging, Visual search, Marginal value theorem, Interruption, Revisitation

## Abstract

In classic visual search, observers typically search for the presence of a target in a scene or display. In foraging tasks, there may be multiple targets in the same display (or “patch”). Observers typically search for and collect these target items in one patch until they decide to leave that patch and move to the next one. This is a highly rule-governed behavior. The current study investigated whether these rules are disrupted when the foraging is interrupted in various manners. In Experiment 1, the foraging was briefly interrupted and then resumed in the same patch. In Experiments 2 and 3, the foraging in each patch either ended voluntarily or compulsorily after a fixed amount of time. In these cases, foraging resumed in a patch only after all patches were visited. Overall, the rules of foraging remained largely intact, though Experiment 2 shows that foraging rules can be overridden by the demand characteristics of the task. The results show that participants tended to perform approximately consistently despite interruptions. The results suggest that foraging behavior in a relatively simple foraging environment is resilient and not easily disrupted by interruption.

## Significance Statement

 "When to quit search" is an important question in visual foraging, especially when the goal of foraging is to achieve the most benefit with limited time and/or energy. While optimal quitting is less critical in everyday search situations, it becomes crucial in expert search situations, such as airport security, medical imaging, and so on. Using the tool of Optimal Foraging Theory, this study examined whether quitting rules remain effective under divided or interrupted foraging conditions. By addressing the resilience of quitting rules in the face of disruptive situations, our study sheds light on the adaptability of visual foraging in a complex world.

## Introduction

Visual search is ubiquitous in everyday human life, with some searches more critical to our well-being than others. For example, the consequence of failing to find Waldo (Klein & MacInnes, 1999) or a typo in this article are relatively innocuous, while those that result from missing a cancerous nodule (Krupinski, 1996) or a fire extinguisher in a burning building (Castel et al., 2012) can be much more dire. The literature on these search tasks is extensive (for a review, see Wolfe, 2023). Most of this research focuses on the question of how targets are found. Less work concerns how and when to end a search. Searches need to end when nothing is found (Becker et al., 2022; Chun & Wolfe, 1996; Zenger & Fahle, 1997). They also need to end when enough is found. This when-to-quit problem is central when one does not know how many targets are present. For instance, in radiology, the problem of “satisfaction of search” arises when the detection of one target encourages searchers to quit before a second target is found (Berbaum et al., 1990, 2018).

When there can be a large number of targets in a display and the goal becomes to collect some or all of them, a search task becomes a “foraging” task. In foraging tasks, a central concern is when to leave the current display (or “patch”; we will use the term “patch”, following the animal literature, though our patches are computer screens full of simple shapes). This quitting or “patch leaving” decision can be seen as representing one aspect of the balance between periods of “exploitation” (collecting targets/resources from one patch) and “exploration” (seeking the next patch) (Cohen et al., 2007). Foraging tasks are common in human and animal life (Stephens et al., 2007). From birds collecting mealworms (Krebs et al., 1977) to humans searching for words in their mental lexicon (Wilke et al., 2009) or foraging for information on the web (Pirolli, 2007; Pirolli & Card, 1999), organisms are continually making decisions about when to stop exploiting the current resource and when to explore for new resources. We used the example of berry picking because the studies reported here use a foraging task loosely modeled on berry picking. In a berry picking task, the typical goal was to collect as much of the resource (the berries) as possible given limited time and energy. In the real world, foragers might move from bush to bush or patch to patch, with the goal of filling their baskets or their mouths with ripe berries. In the simulated berry picking task from the lab, those berries were on-screen patches of color. Moving from patch to patch takes time and/or effort. During that “travel time”, target collection was not allowed. Nevertheless, foragers travel in anticipation of arrival at a new patch where new, collectable targets are expected. Considering this trade-off, foragers typically leave the current patch/screen at some point before complete exhaustion of the current resource. Foragers tend to leave when the yield from the current patch has declined to the point that the foragers decide that it is worth taking the cost of travel.

Foraging behaviors are widely discussed in the animal literature (e.g., Bond, 1983; Hahn et al., 2005; Stephens & Krebs, 2019) and there is an increasing body of work on foraging in humans performing a range of tasks from our berry picking task (Jóhannesson et al., 2016; Kristjánsson et al., 2020; Wolfe, 2013) to the aforementioned memory foraging (Wilke et al., 2009). Human foraging behavior is broadly similar to animal foraging (e.g., Louâpre et al., 2010; Wolfe, 2013) but see Stephens & Dunlap (2009). The basics of human foraging behavior are present early in development and may be innate or gene-driven (Crittenden et al., 2013; Pretelli et al., 2022).

There is a rich array of approaches to modeling the components of foraging behavior (Bella-Fernández et al., 2022; Clarke et al., 2022a, 2022b). For modeling the patch-leaving/quitting time aspect of foraging, one appealing account is optimal foraging theory (Pyke et al., 1977), as embodied in the “marginal value theorem” (MVT; Charnov, 1976). MVT holds that a forager should leave the current patch when the instantaneous rate of return from that current patch drops to the average rate of return for the task as a whole. This average rate includes the travel time between patches. Travel time drives down the average rate since you cannot collect berries between patches.

In the present study, we used MVT-style analysis to investigate how foraging behavior was impacted when the foraging was *interrupted*. For example, imagine that you are picking berries when your foraging is interrupted by a phone call and that you stop collecting to concentrate on the call. This reduces your average rate of berry collection. With that lower average rate, you should pick in the current patch for longer until your current rate of collection falls to this lower average rate. Alternatively, you might discount that phone call, removing those 30 s from the calculation of the average rate. We can also ask what happens when the phone call ends and you return to foraging. When foraging is interrupted or split for whatever reason, you might *revisit* some places that you have previously foraged after a period of being elsewhere. To give an example of a situation more consequential than berry picking, how does an interruption change the behavior of a doctor foraging for signs of the spread of cancer in an abdominal CT scan? In most previous foraging research, patches are visited only once and revisitation is not allowed. We do not know how human foragers respond when they return to a patch that has been partially foraged.

We are focusing on discrete periods of interruption that take the forager away from a task. We can distinguish this from “interference” where something occurs that might slow or alter ongoing foraging behavior. In the human literature, a good example of interference is found in the work of Thornton et al (2021). In their paradigm, foraging human “sheep” needed to keep an eye out for “wolves” who might suddenly turn hostile. The vigilance required to avoid predators altered foraging behavior, making it more likely, for example that observers would switch from one type of foraging target to another.

In other tasks, interruption tends to have negative consequences, ranging from simple task-switching costs (Monsell, 2003; Rogers & Monsell, 1995) to potentially hazardous accidents (Borowsky et al., 2016). Anecdotally, experts like radiologists report being concerned about the ill effects of interruption. Interestingly, some empirical studies have found that sudden interruption in visual search did not impair accuracy, though it did extend reaction time (Drew et al., 2018; Radović et al., 2022; Williams & Drew, 2017). However, the interruption costs did increase when a search task with a high working memory demand was interrupted (Alonso et al., 2021).

In other settings, revisiting a display produces positive outcomes. Repeatedly searching for a target within an identical distractor layout can reduce target detection time (Chun & Jiang, 1998) and/or reduce the possibility of missing the secondary target (Cain et al., 2014). Double-reading in radiology improves performance, reducing misses (Karmazyn et al., 2017), presumably, at least in part, because having two independent detectors produces benefits due to probability summation. It would be interesting to ask if giving one observer two chances to visit the same image will also produce benefits in a foraging setting. Will participants stay for a longer/shorter time and/or forage more successfully for berries if they can return to patches that they have already visited?

As noted, we will look at the effects of interruption using the tools of MVT. The MVT approach to analyzing foraging has some known limitations. It relies on comparison between the instantaneous rate of return and the average rate. “Instantaneous” rate is problematic in many foraging situations. For example, a lion may engage in periods of exploration and exploitation, but here “exploitation” is the process of eating prey. The hunting or “exploration” time starts, not when the instantaneous rate of eating drops below the average rate, but when the lion gets hungry or, perhaps, when the lion “realizes” that it will be hungry if another antelope is not collected. In cases like this, the problems are similar to those of other foraging scenarios but the MVT rules are not as directly applicable. In general, simple MVT accounts become problematic in tasks where the rate of return drops to zero for extended periods of time. For example, Ehinger and Wolfe (2016) had observers look for gas stations in aerial imagery. One solution is to adopt a Bayesian version of an MVT-style analysis in which “instantaneous rate” is replaced by something like an “expected rate” (Cain et al., 2012; Ehinger & Wolfe, 2016). The MVT use of an average rate of return is also problematic because that average rate must be learned (Constantino & Daw, 2015; Davidson & El Hady, 2019; Harhen & Bornstein, 2023; Kolling & Akam, 2017) or sometimes it is discounted (Kagel et al., 1986). It also must be possible to update that rate estimate in response to changes in a dynamic search environment (Fougnie et al., 2015; Wolfe et al., 2018; Zhang et al., 2017). Patch-leaving is sometimes modulated by biological (Le Heron et al., 2020; Struk et al., 2019) or developmental (Lloyd et al., 2023; Wiegand et al., 2019) aspects of animal (Clarke et al., 2022a, 2022b; Irons & Leber, 2016, 2018; Á. Kristjánsson et al., 2014).

The nature of the travel time also complicates many real-world foraging tasks. The lion, mentioned above, may need to decide between venturing out on a short trip to capture small prey and a longer one to find more rewarding, larger prey (Chittka et al., 2009). Levy flights/walks have been one popular way to model this “exploration” phase of foraging (Bella-Fernández et al., 2022; Garg & Kello, 2021; Raichlen et al., 2014) (for an alternative, see Nolting et al., 2015).

With all of these caveats, one might wonder if Optimal Foraging Theory is useful at all (Clarke et al., 2022a, 2022b; Kolling & Akam, 2017; Pierce & Ollason, 1987). Certainly, there are plenty of reports of behavioral deviation from optimality in animals (e.g., Carter & Redish, 2016) and humans (e.g., Le Heron et al., 2020). Nevertheless, it seems rash to agree with Pierce and Ollason’s (1987) assertion that “optimal foraging theory is a complete waste of time.” In a task like berry picking (real or on-screen), resources are acquired more or less continuously, making instantaneous rate a meaningful quantity. The task is stable over time, making the average rate also meaningful, even if it does, obviously, need to be learned and monitored. In our version, travel time is fixed and the travel delivers the forager reliably to a new patch of berries. This, perhaps, simulates an implausibly regular field but it does essentially eliminate the variability in the exploration stage.

Within this somewhat idealized berry patch, it is possible to use MVT to examine the effects of interruption on foraging behavior. Moreover, the components of the MVT calculations, instantaneous rate, overall rate, etc., remain interesting metrics of performance, even without a commitment to MVT. In that spirit, in Experiment 1, we compared conditions where foraging was briefly interrupted and resumed to conditions where it was not interrupted. In Experiments 2 and 3, the foraging in each patch was stopped, either by an involuntary time limit or by participants’ voluntary choice to stop. Then, having visited all patches, participants revisited the patches. The targets collected and the patch-leaving strategy were compared with those of uninterrupted foraging. To anticipate our main results, in these experiments, foraging behavior was not significantly disrupted by interruption.

## Experiment 1

### Method

#### Participants

Sample size was based on Wolfe (2013). In those studies, 10 observers were adequate to produce reliable measures of the relationship of instantaneous and average rates. We planned to double the sample size in order to increase the statistical power. A total of 22 participants were recruited from Prolific (https://www.prolific.com/). One participant was excluded from the analysis because this participant tended to leave patches so early that they were rarely interrupted in our design. Participants submitted an online consent form before the experiment started. The procedures were approved by the Institutional Review Board at Brigham and Women’s Hospital.

#### Stimuli

**Fig. 1:**
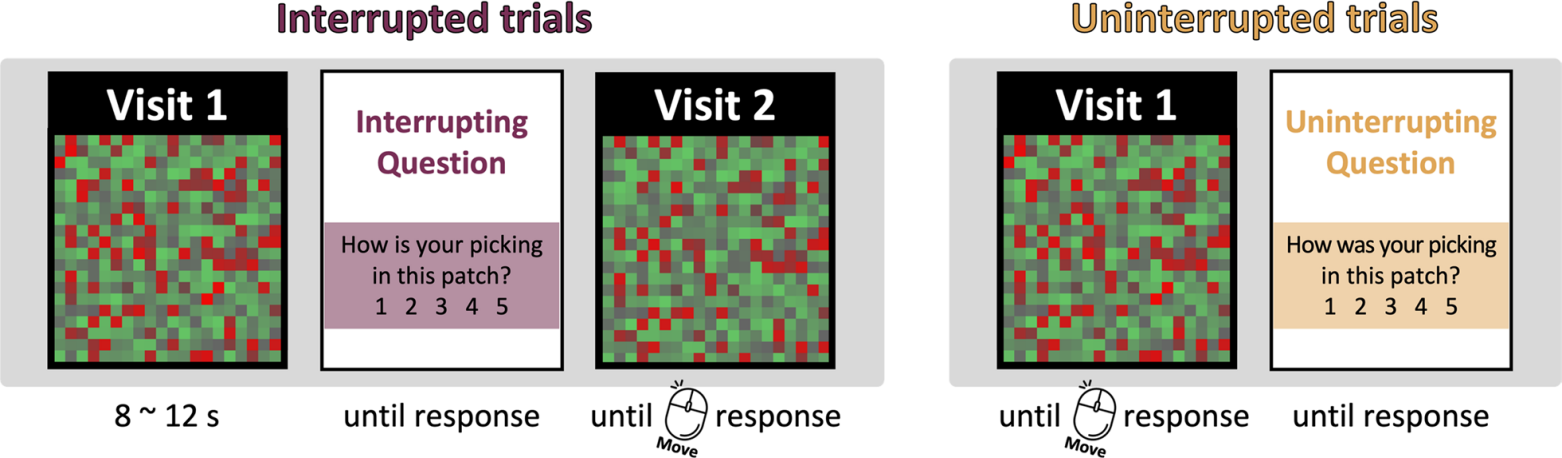
Schematic description of procedures for Experiment 1: On Interrupted trials, participants foraged for 8–12 s before being interrupted to answer a question about the quality of picking. Foraging then resumed in the same patch as “Visit 2” until terminated by the participant’s patch-leaving click. On Uninterrupted trials, participants foraged until they chose to leave the patch, without being interrupted. After the patch-leaving click, they were asked the same question as in Experiment 1

The experiment was programmed with PsychoPy and PsychoJS library, and was run on Pavlovia (https://pavlovia.org/). Participants foraged through a series of on-screen “berry patches” as shown in Fig. 1. The task was performed online so we did not have control over the exact stimulus size or the viewing distance. Participants were constrained by experimental conditions so they couldn't initiate the experiment using mobile phones or tablet PCs. Patches were sized to be 0.7 of the maximum screen height. Each patch was composed of 20 × 20 colored squares. Out of these 400 colored squares, 20, 25, or 30% of the squares were red “berries.” Half of those berries were “good” targets (ripe berries), while the other half were “bad” targets (unripe berries). The color of each berry was defined by the triplet [R, (255-R)/2, (255-R)/2] in RGB color space (R means the value of the red color channel). R for the good berries were randomly selected from a normal distribution with a mean of 200 and a standard deviation of 20. The colors of the bad berries were sampled from another normal distribution with a mean of 150 and a standard deviation of 20. Thus, the “good” and “bad” distributions overlapped, making a perfect performance virtually impossible. The best possible performance would have a d’ of (200–150)/20 = 2.5. Participants were instructed that the bright red squares were ripe and good berries while dimmer red squares were bad berries. Non-berry squares were green “leaves,” whose color was defined in the RGB color space by the triplet [100, G, 100], where G stands for the value of green channel sampled from a uniform distribution between 100 and 200. The patch number (Patch # X out of 30; X refers to the patch number) was presented on top of the patch.

During the period between picking in one patch and the next, participants viewed an animation on screen meant to simulate travel. A white rectangle of the same size as the patch in the x-dimension was positioned at the center of screen. Bushes, sized 0.15 of patch, were at the left and right side of travel path, respectively. A basket moved from left to right, filling the path with a yellow bar, which grew proportional to the elapsed time from the travel onset.

#### Procedure

Participants were given a “field”, or a block, with 30 patches and were instructed to click as many good berries as possible. When the berry was clicked, the square turned into a greenish “leaf”, accompanied by “good” or “bad” sound feedback. After participants collected as many targets as they wished in a patch, they clicked the ‘next’ button on the right side to move to the next patch. The next patch was presented after a two-second “travel time” delay. Participants watched the basket on the travel path cross the screen. Participants could not pick berries during this travel time. When the basket reached the right end, the travel time was over, and the following patch began. The entire experiment lasted approximately 20–30 min.

Whether foraging would be interrupted or not was randomly determined every trial. A patch in which foraging was interrupted will be thus referred to as an ‘interrupted’ trial. On interrupted trials, the question “How is your picking in this patch?” appeared on the screen 8, 10, or 12 s after patch onset, depending on the set size. A response was made on a five-point scale (1: terrible to 5: great). Participants clicked one of the scores and then pressed the ‘space bar’ to resume foraging in the same patch. Picking in the current patch ended if participants made the patch-leaving decision before the question popped up. In ‘uninterrupted’ trials, the same question (in the past tense)—“How was your picking in this patch?”—was presented immediately after participants decided to leave the current patch and right before the travel began.

### Results

Does splitting foraging in a patch into two sections affect the quantity and quality of gain? Participants spent, on average, 2.16 s (SD = 1.18 s) answering the question shown in the interrupted patch and 2.99 s (SD = 2.14 s) in the uninterrupted patch. It is worth mentioning that this interruption is much shorter than the interruptions used in previous interruption studies: e.g., 20.6 s (Alonso et al., 2021) and 38.2 s (Williams & Drew, 2017).

**Fig. 2:**
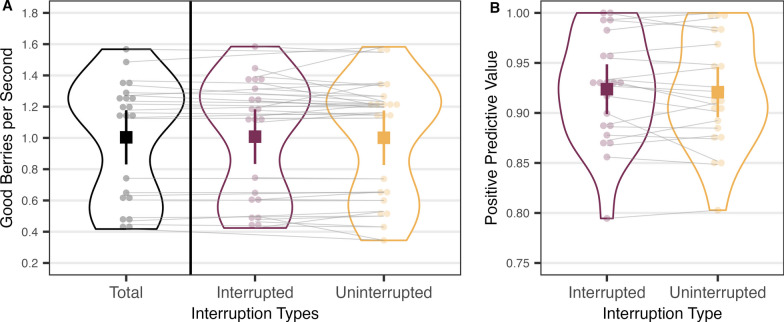
**A** The overall rate of return and **B** positive predictive value from Experiment 1. The graphs show individual observer data points (transparent circles) and summarized data points (solid squares). Error bars are 95% confidence intervals. Grey lines connect the data points from the same participant

We removed 3.18% of trials as they had reaction times that were slower than 4 s or faster than 200 ms. Participants gave the picking quality an average rating of 2.88 (SD = 0.88) for the interrupted trials and 3.36 (SD = 0.55) for the uninterrupted trials. Participants rated uninterrupted trials more favorably, *t*(20) = −2.766, *p* = 0.011. We conducted non-parametric statistical tests for observations that did not meet the basic assumptions of the parametric tests. Descriptive statistics are shown in Table 1, and a graphical description of yield is in Fig. 2. Looking at Table 1, note that the sum of the first and second intervals for each measure in the interrupted condition is very similar to the total in the uninterrupted condition for clicks per patch, hit rate, and false alarm rate.

**Table 1:** Mean (standard deviation) descriptive information for Experiment 1

Interruption type	Patches visited		Clicks per patch		Average hit rate		Average FA rate	
	First	Second	First	Second	First	Second	First	Second
Interrupted	14.95 (0.22)	12.43 (4.20)	13.48 (5.73)	19.60 (13.70)	0.25 (0.10)	0.34 (0.22)	0.01 (0.01)	0.05 (0.05)
Uninterrupted	14.86 (0.48)		31.58 (15.91)		0.57 (0.28)		0.06 (0.06)	

“First” and “Second” refer to the foraging periods before and after interruption. Hit = proportion of picked berries that were “good”. FA = proportion of picked berries that were “bad”

First, we examined how many good berries foragers obtained on average during the interrupted and uninterrupted trials. We divided the number of good berries harvested by the time spent in each interruption type, including the time spent on the question and the travel time between patches, as is shown in Fig. 2A. A Wilcoxon signed-rank test showed that participants obtained a similar quantity of berries from the interrupted and uninterrupted patches, *V* = 127.00, *p* = 0.708.

Second, we examined whether the quality of foraging was affected by interruption by comparing the positive predictive value (PPV) of clicks; that is, the probability that a forager’s click would produce a positive outcome (Fig. 2B). As good and bad targets had overlapping color distributions, participants could not perfectly discriminate between them. Therefore, even though the foragers’ goal was to maximize the hit rate (correctly clicking on good berries), they sometimes committed false alarms (clicking on bad berries). PPV is defined as hits divided by the sum of hits and false alarms. As with the overall rate of return, PPV was not significantly different across the interruption types, *t*(20) = 0.839, *p* = 0.411.

Third, we tested whether interruption had an impact on patch-leaving time. The interruption might be seen as lowering the yield for that patch, predisposing participants to quit rapidly when the foraging resumed after the interruption. Alternatively, the interruption could be seen as irrelevant to foraging behavior and ignored for patch-leaving determinations. Each click generates a response time (RT) and either does or does not add to the resources collected by the participant. To aggregate those berries and RTs across patches and across observers, it is useful to align clicks to the *final* click in the patch, the moment of patch leaving. Thus, to obtain the instantaneous rate of return, the time per click was averaged in reverse order from that final click within a patch. For example, if participants made 10 clicks in a patch, the final, 10th click would be the first “reverse click”. The ninth click would be the second reverse click and so forth. PPV is calculated as a function of reverse click. The instantaneous rate of berry intake (good berries per second) is obtained by dividing the average PPV by average RT at each click position. As patches are depleted, PPV typically decreases and RT increases. This produces a decreasing instantaneous rate of return. In this calculation, the first clicks in forward sequences are excluded because those RTs are markedly slower than other clicks, presumably due to visual processing costs of stimulus onset.

**Fig. 3:**
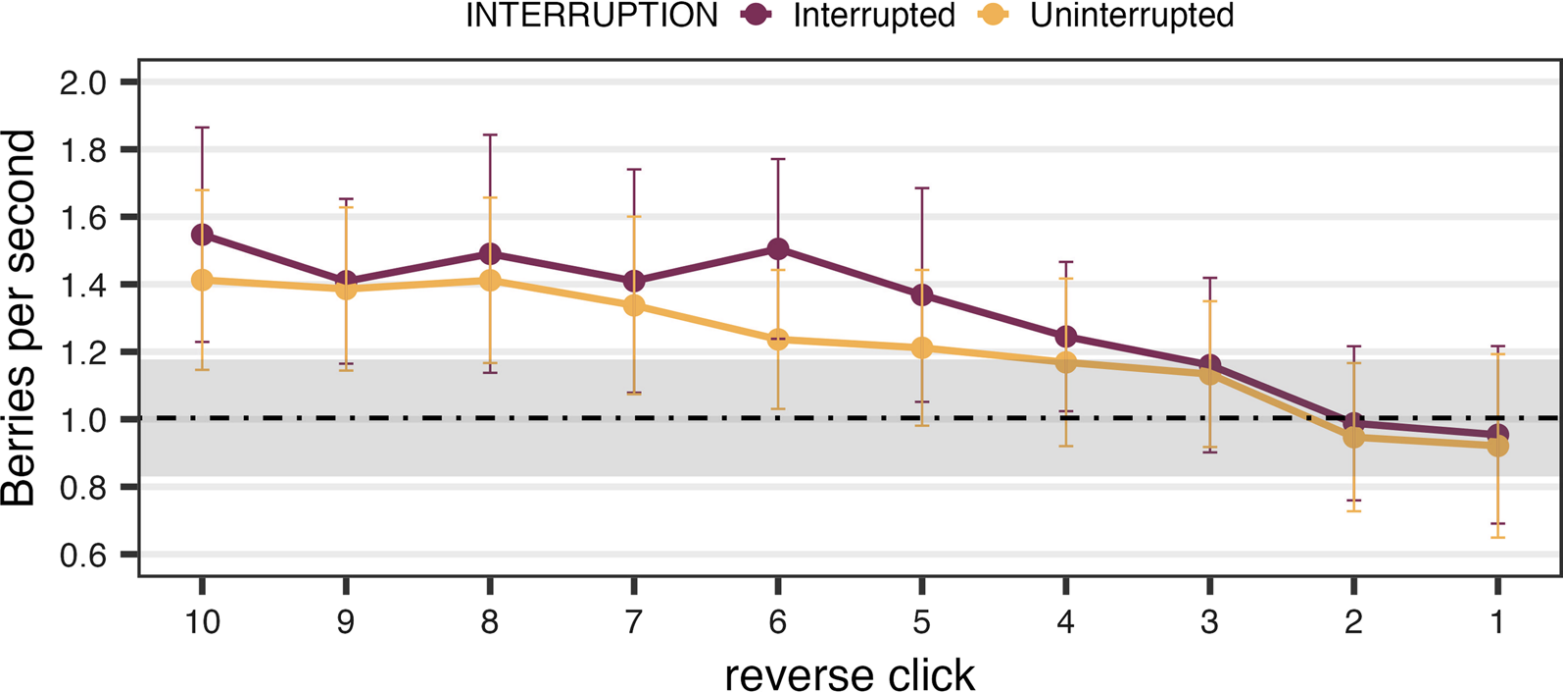
Instantaneous rate of return as a function of reverse click order from Experiment 1. *Note*. Horizontal black line indicates the average rate of return, and shaded area is the 95% confidence interval of the average rate. Error bars around each mean instantaneous rate represent 95% confidence intervals

Figure 3 depicts the instantaneous rates of return as a function of the reverse click sequence. For the interrupted trials, only the clicks that happened after the interruption were included. It can be seen that the patch-leaving behavior is broadly optimal from the MVT’s perspective and essentially the same for interrupted and uninterrupted patches. Simply speaking, participants left the interrupted and uninterrupted patches when the instantaneous rates dropped down to the overall rate (Table 2). Pairwise *t*-tests on the five last clicks show that the instantaneous rate is greater than the average then declines to a level indistinguishable from the average rate for last few clicks. This MVT-style patch-leaving was found in both the interrupted and uninterrupted trials. The results imply that participants adopted a similar patch-leaving strategy despite a brief interruption in the middle of active foraging.

**Table 2:** Total rate and instantaneous rates of return from each reverse click order in Experiment 1

Interruption type	Total rate (SD)	Click 5	Click 4	Click 3	Click 2	Click 1
Interrupted	1.01 (0.38)	1.37*	1.25*	1.16	0.99	0.95
Uninterrupted	1.00 (0.38)	1.21*	1.17	1.13	0.95	0.92

^*^p < .05. *p* values from the interrupted and uninterrupted trials were adjusted using the Bonferroni method for multiple comparisons

### Discussion

Experiment 1 tested the possibility that interruption by an irrelevant event would undermine foraging optimality as defined by MVT. However, patch-leaving behavior was approximately the same and approximately equally optimal in the interrupted and uninterrupted conditions. Participants picked similar numbers of berries in similar ways whether they were interrupted or not. Interruption did not interfere with their implicit marginal value calculations.

## Experiment 2

In Experiment 1, foraging was temporarily interrupted by a question, and participants then resumed berry picking in the same patch as soon as the question was answered. Participants could have developed an expectation that visual foraging would be interrupted intermittently and that foraging would then resume despite interruption. Experiment 2 examined the situation in which foraging trials are interrupted one patch after another and then unexpectedly resumed only after multiple patches were visited once. This situation is like taking a quick tour through an art museum and then finding that there is time to revisit locations in a more leisurely fashion once everything was looked at once. Would the initial visit to a site influence the patch-leaving strategy when participants revisited it?

In the interrupted groups, participants first visited all the given patches in a field once and then sequentially revisited the patches. Many patches intervened between the first and second visits to a patch. The two interruption groups differed in the nature of the interruptions. In the Forced Move group, foraging was interrupted by participants being moved from one patch to another by the experimenters. In the Choice Move group, the participants left the patch when they wished and were subsequently returned to the patch for a second round of foraging.

### Method

#### Participants

We recruited 65 participants (Forced Move *N* = 20, Choice Move *N* = 25, Control *N* = 20) from Prolific. We originally planned on recruiting 20 participants per group, as per the sample size estimation in Experiment 1.

#### Procedure

**Fig. 4:**
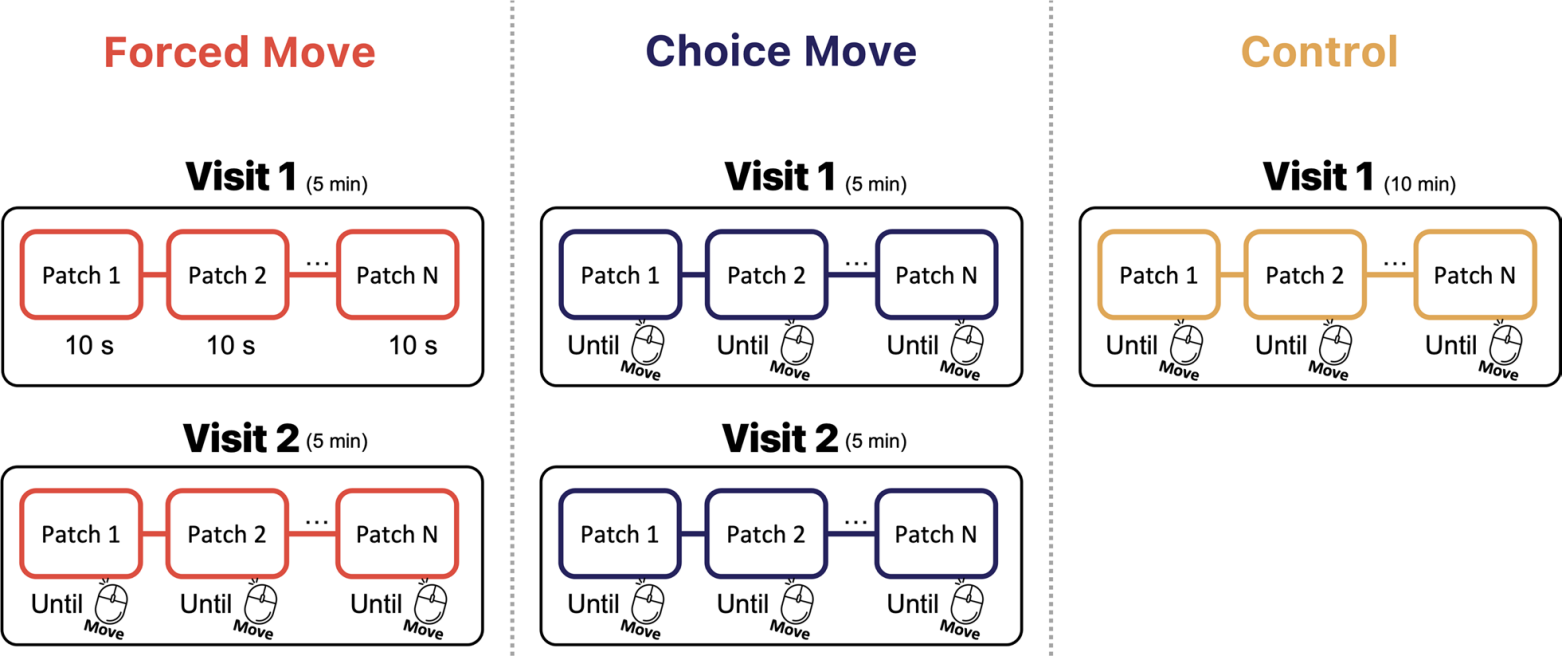
Cartoon of the three conditions in Experiments 2 and 3. In the Forced Move condition, Visit 1 ended after 10 s in Experiment 2 and after 8, 10, or 12 s in Experiment 3. In the Choice Move condition, each initial visit to a patch ended when participants wished. In the Control condition, participants left each patch when they wished. Note that in Experiment 3, time pressure was added to the initial visit of Choice Move condition as participants were told to leave when they wished BUT to try to get to all of the patches in 5 min

Participants were randomly assigned to one of the three conditions (Forced Move, Choice Move, or Control). In the Forced Move and Choice Move conditions, participants completed two visits to each patch in a fixed, sequential order. Each phase took 5 min, so the entire task took a total of 10 min. The control condition consisted of a single pass through the patches, lasting 10 min without a break. A schematic description of the procedure can be found in Fig. 4.

The patch displays and the foraging task were similar to Experiment 1. Set sizes could be 15, 20, 25, 30, or 35% of the squares in the 20 × 20 array. A white time bar of the same length as the patch was positioned on top of the patch. The bar filled with yellow from left to right proportionally to the elapsed time from the experiment onset until the given time per patch (Forced Move condition) or field (Choice Move and control conditions) is up.

In the first visit for the Forced Move condition, the time limit was 10 s per patch. After those 10 s, foraging in a patch stopped and participants were forced to travel to the next patch. The first visit portion of the task was over when a participant had visited all 25 patches in the field.

In the Choice Move condition, participants visited as many patches as they wished and foraged for as long as they wanted within an overall 5-min limit. There was no restriction on the number of patches, or the time spent for each patch. Participants could proceed to the next patch at any time by clicking the ‘next’ button. Two-seconds of travel time delay were interjected between patches.

Participants in the Forced Move and Choice Move conditions were not told that there would be a second chance to visit the patches. However, after the 5-min first set of visits, a surprise instruction appeared on the screen, saying that participants were allowed to revisit the patches in a field for extra 5 min so that they could collect targets that they might have missed on their first visit. The patches that participants had scanned were provided again in the original order they appeared in the first visit. Each patch started in the state where it had been when the target collection stopped on the first visit. In the second visit, all participants in both interrupted groups could proceed to the next patch whenever they wanted. Participants were instructed to collect as many good berries as possible in 5 min. The second visit was terminated when the 5-min limit was reached, even though participants might have visited fewer patches than the number of patches foraged during the first visit. The second visit could end earlier than 5 min if a participant visited all of the patches that they had visited in the first round prior to then. The time bar reset at the first patch onset and grew again proportionally until 5 min were up.

In the Control condition, the experiment consisted of a single visit. Participants were given 10 min to forage through as many patches as they wished with a 2-s travel time between each patch. Thus, in the Control condition, patches were foraged only once.

### Results

*Data exclusion:* We excluded two participants (one from the Forced Move group and one from the control group) whose average RT exceeded 4 s per item. The mean RT across participants before this exclusion was 1.35 s (SD = 2.49 s) and after exclusion 1.00 s (SD = 0.39 s). Next, we excluded the 2.04% of trials with RTs longer than 4 s or shorter than 200 ms. Finally, in the Forced and Choice Move conditions, the patches that were not foraged twice were excluded from most analyses (except for the overall rate of return; see below) because those patches do not meet our research goal of examining the quitting decision on the second visit to the patch. The number of participants, the average number of scanned patches, clicks, hit and false alarm rates from each condition are given in Table 3.

**Table 3:** Mean (standard deviation) descriptive information for Experiment 2: “First” and “Second” refer to the foraging periods before and after interruption

Interruption type	Patches visited	Clicks per patch		Average hit rate		Average FA rate	
		First	Second	First	Second	First	Second
Forced Move	12.32 (4.61)	11.46 (3.88)	22.63 (5.74)	0.28 (0.10)	0.42 (0.10)	0.01 (0.02)	0.06 (0.03)
Choice Move	8.60 (3.71)	34.05 (10.61)	15.44 (5.60)	0.67 (0.16)	0.20 (0.11)	0.08 (0.06)	0.13 (0.07)
Control	17.05 (4.27)	36.61 (9.03)		0.74 (0.13)		0.09 (0.05)	

Patches visited = Number of patches visited twice for Forced Move and Choice Move groups, once for the Control group. Hit = proportion of picked berries that were “good”. FA = proportion of picked berries that were “bad”. Again, notice that the sum of the first and second patches can be compared to the totals for the control condition

#### Overall performance

**Fig. 5:**
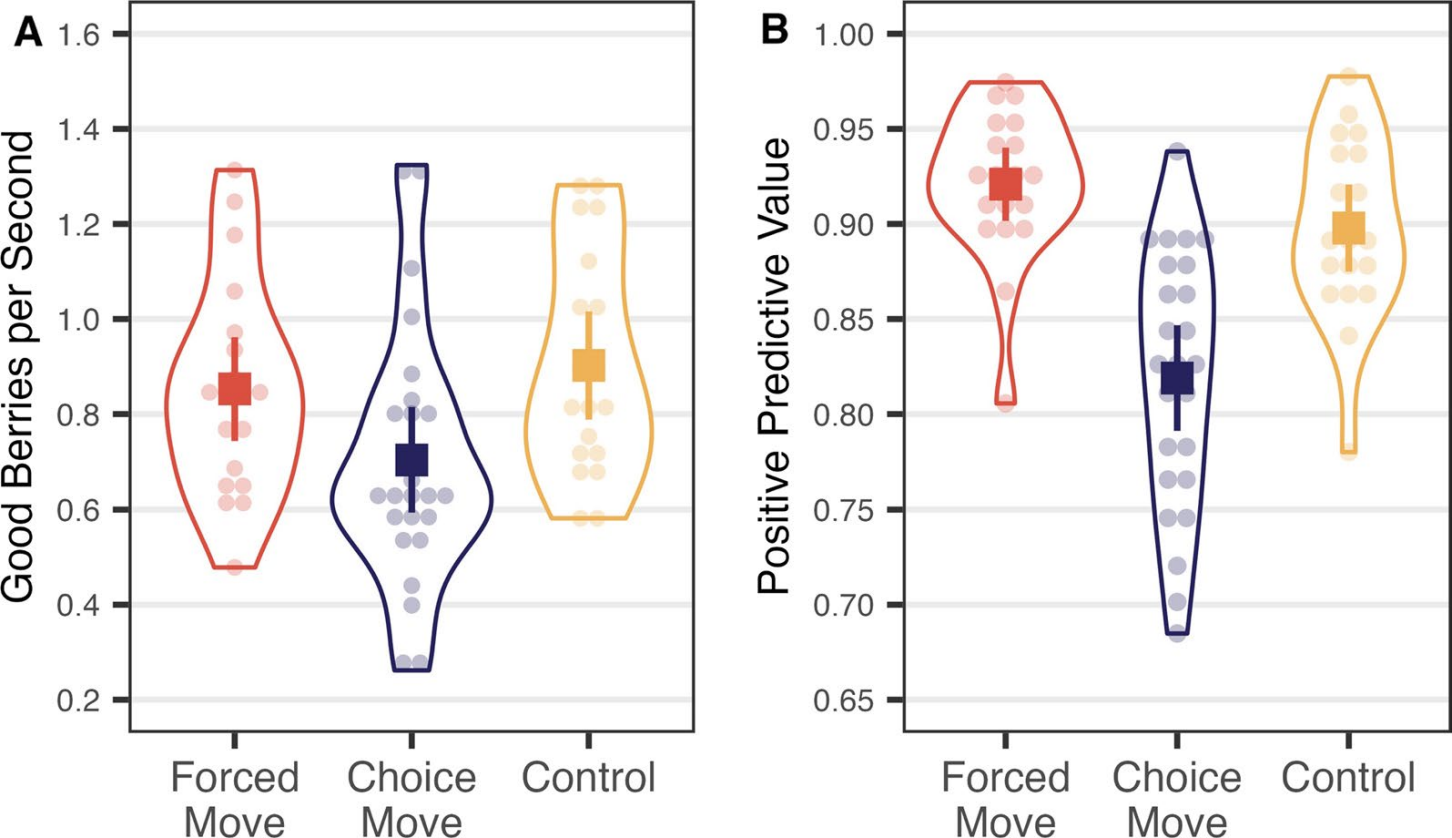
**A** Overall rate of return and **B** PPV of Experiment 2: *Note*. The graphs show individual data points (lighter circles) and averaged data points (solid squares). Error bars are 95% confidence intervals

First, we examined how many good berries foragers obtained on average during the entire foraging experience. This includes time spent in each patch and travel time, but does not include a break between visits with a surprise instruction. As the overall rate of return reflects the total gain, we used all the patches including single-scanned patches from the Forced and Choice Move conditions in calculating it. Figure 5A shows the overall rate of return from each condition. It can be seen that the Control and Forced Move conditions are similar to each other, while the Choice Move condition produces apparently worse performance; an odd result that is less odd on closer examination, as discussed below. A Kruskal–Wallis test showed that the overall rates of return differed by condition, *χ*^2^(2) = 8.507, *p* = 0.014, *ε*^2^ = 0.137. Dunn’s test with the Benjamini–Hochberg correction method showed the Choice Move condition resulted in a significantly smaller gain: Choice–Control *z* = −2.682, *p* = 0.022; Choice–Forced *z* = −2.192, *p* = 0.043. The average yields in the Forced Move and Control groups were not different: Forced–Control, *z* = 0.460, *p* = 0.645.

Second, we tested the proportion of good berries among all berries picked, or PPV (Fig. 5B). As with the overall rate of return, PPV differed across condition, *χ*^2^(2) = 30.319, *p* < 0.001, *ε*^2^ = 0.489. Looking at the pairwise comparisons, PPV was smallest in the Choice Move condition: Forced–Choice *z* = −5.440, *p* < 0.001; Choice–Control *z* = −3.119, *p* = 0.003. The Forced Move condition resulted in a higher PPV than Control condition, *z* = −2.177, *p* = 0.029.

The primary driver of these differences can be seen in Table 2. Looking at the Clicks Per Patch, we see that participants gathered about 37 berries per patch in the control condition. When foraging was forcibly interrupted, participants returned to the patch and collected enough berries to get to an average of 34 berries per patch in total, comparable to the Control condition. In the Choice Move condition, however, participants gathered those 34 berries per patch on their first visit due to lack of knowledge that they would later revisit the patches. When unexpectedly given the chance to continue foraging, they presumably concluded that they were expected to do something with their time and collected another 15 berries per patch. The remaining berries are harder to acquire and easier to confuse with bad berries, driving down the rate of return and the PPV. As we will see below, the rules of the Choice Move condition persuaded the participants to apparently overharvest the patches on their second visit.

#### Different conditions produce different response criteria

**Fig. 6:**
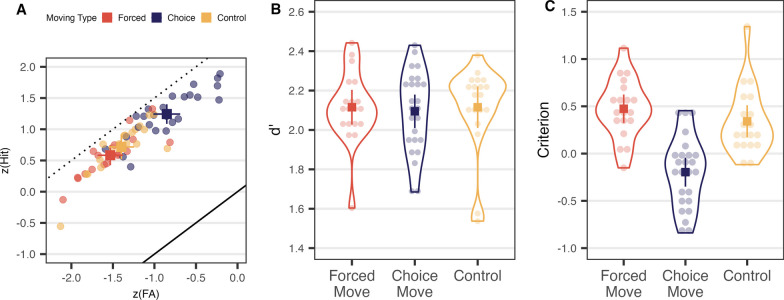
Signal detection measures for Experiment 2: **A** Receiver operating characteristic on z-coordinate axes. Lighter, semi-transparent dots are individual observers, and solid squares show average results. Error bars are 95% confidence intervals. The solid diagonal line indicates expected *z*(Hit) and *z*(FA) when *d*′ is 0 (chance). The dotted diagonal line indicates expected *z*(Hit) and *z*(FA) if *d*′ was the maximum possible 2.5. **B** *d*′ for each condition. **C** Response criterion for each condition

Figure 6 shows that the main effect of condition in Experiment 2 is on decision criterion. Figure 6B shows that there is no significant effect on *d’*, *χ*^2^(2) = 0.423, *p* = 0.809, *ε*^2^ = 0.007, while Fig. 6C shows that there is an effect on criterion, *F*(2, 60) = 23.267, *p* < 0.001, *η*^2^_*p*_ = 0.437, with the Choice Move condition producing a more liberal criterion than the Control or Forced Move conditions, Choice—Control *t*(60) = −5.057, *p* < 0.001; Choice-Forced *t*(60) = −6.313, *p* < 0.001; Control-Forced *t*(60) = −1.178, *p* = 0.730. This result is summarized in Fig. 6A where the results are plotted on the receiver operating characteristic on z-coordinate axes. The results for most participants in all conditions fall a bit below the straight line that would be produced by the best possible performance (*d'* = 2.5). The liberal shift in criterion in the Choice condition is seen as a shift in data points up and to the right as participants produce more hits and more false alarms.

#### Quitting strategy in multiple foraging

**Fig. 7:**
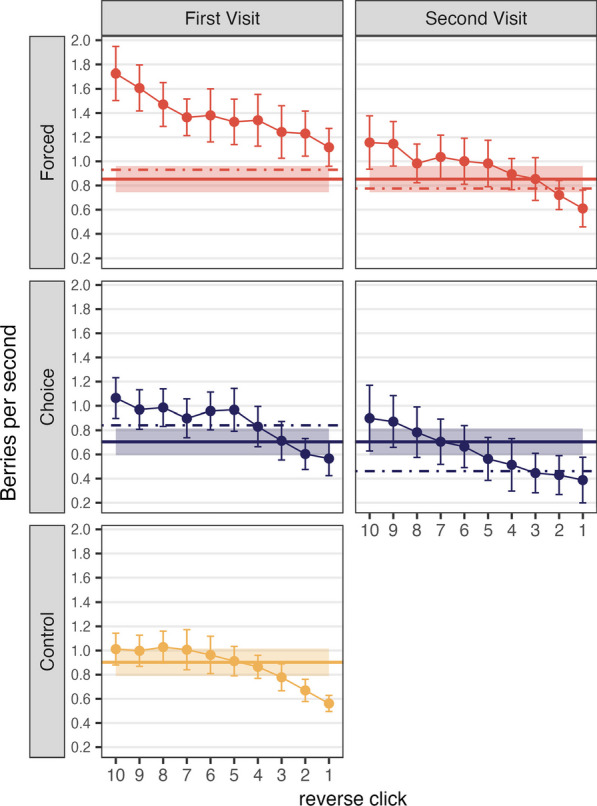
Instantaneous rate of return, or expected number of berries per second, as a function of reverse click order in Experiment 2. Error bars depict 95% confidence intervals. Solid horizontal lines are the average rates of return from the entire habitat, and shaded areas are 95% confidence intervals. Dashed lines are the average rate of return from each visit

In the Control condition and in the second round of foraging of the Forced and Choice Move conditions, participants terminated foraging from the patches at their own pace. As before, we obtained instantaneous rates of return by dividing PPV by RT as a function of reverse click position. Figure 7 shows the instantaneous rate of return for the last 10 clicks in the patch. The solid horizontal line shows the overall rate across the entire task. The dashed lines show the overall rate calculated separately for the first and second portions of the task. The Control condition only has one portion.

MVT predicts that participants will leave the patch when the instantaneous rate drops below the average rate. Here, we observed that, in the Control condition and the second part of the Forced condition, participants’ patch-leaving is close to the MVT prediction with some evidence of “overharvesting” (staying longer in the patch than predicted by MVT), a phenomenon that occurs with some frequency in the foraging literature (Carter & Redish, 2016; Constantino & Daw, 2015; Hayden et al., 2011; Kane et al., 2022). As shown in Table 4 and Fig. 7, the Choice Move condition showed more dramatic overharvesting. For the Choice Move condition, the last five clicks are significantly below the average rate for the task.

**Table 4:** Total rate and instantaneous rates of return from each reverse click order in Experiment 2

Moving type	Total rate (SD)	Click 5	Click 4	Click 3	Click 2	Click 1
Forced Move	0.85 (0.23)	0.98	0.89	0.85	0.72*	0.61**
Choice Move	0.70 (0.27)	0.56*	0.51**	0.45***	0.43***	0.39***
Control	0.90 (0.24)	0.91	0.86	0.78	0.67***	0.56***

^*^p < .05, **p < .01, ***p < .001. *p* values were adjusted using Bonferroni correction method

### Discussion

The results of Experiment 2 indicate that forcibly interrupting foraging did not markedly change foraging behavior. When given the opportunity to revisit patches that they had been forced to leave, participants behaved much as they behaved in the Control condition where foraging was uninterrupted. In the Forced condition, participants are forced out of multiple patches and then allowed to revisit them. It is not likely that participants remembered the state of specific patches. More plausibly, when returned to somewhat depleted but still fertile patches they based their behavior on their assessment of the current situation and left the patch when the returns were sufficiently diminished.

Behavior seems somewhat different in the Choice Move condition. The Choice Move group forages for longer than other two groups. Here, participants were allowed to pick as long as they wanted during their first visit to the patch. Reasonably enough, they treated that first epoch like the Control condition, but then they were unexpectedly required to revisit the patches again. This can be seen by comparing the statistics for Control and Choice (first visit) in Table 3. As a result, when, to their surprise, they were asked to forage in those patches again, they would seem to have little or no reason to forage further on that second visit. However, they do keep foraging. There are at least two possible accounts for this behavior. One possibility is that the perceived demands of the experimental situation inclined participants to collect more targets from each depleted patch. As a result, they picked more mediocre berries and ended up harvesting past the MVT-optimal moment of departure as seen in Fig. 7. This is seen as a criterion shift to a more liberal position in Fig. 6. Another possibility is that participants switched to a new, lower estimate of the overall rate. When they were sent back to pick again in the previously picked patches, it was harder to find new good berries. The resulting lower overall rate for the second part of the task is shown as the dashed line in Fig. 7. It can be seen that participants leave the depleted patch at a roughly MVT-optimal point, if we imagine that they were using this lower overall rate (see Fougnie et al., 2015).

## Experiment 3

As noted, it is possible that the Choice Move condition of Experiment 2 differed from the Control and Forced Move conditions only because participants felt obligated to do some foraging in order to comply with the perceived demands of the experimenters, even if that violated the predictions of the MVT. In an effort to change the demand characteristics of the Choice Move condition, Experiment 3 repeated the conditions of Experiment 2 but with different Choice Move condition instructions that encouraged participants to leave patches more quickly in their first visit. The goal was to leave the participants with some berries worth picking when they returned for the second visit.

### Method

#### Participants

We collected 60 valid data sets from Prolific. The experiment took about 15–20 min. The procedures were approved by the Institutional Review Board at Brigham and Women’s Hospital.

#### Procedure

The procedure is cartooned in Fig. 4 and is essentially the same as Experiment 2 with one important change to the Choice Move condition. There were 25 patches available to forage. In this version of the Choice Move condition, participants could leave a patch whenever they wished, but they were told that they were expected to visit at least 20 patches and, ideally, to visit all 25, in the first phase of the experiment. This instruction was intended to persuade participants to leave each patch when there were still items worth collecting. In the Forced Move condition, participants were automatically moved to the next patch after 8, 10, or 12 s when the set size was 80, 100, and 120 berries (40, 50, and 60 “good berries”), respectively. This timing meant that participants from the Forced Move and Choice Move conditions spent comparable time in each patch during the first phase and that they left sufficient berries behind in both conditions to make further foraging worthwhile. The first visit finished after 5 min or when all 25 patches in a field were visited. In the Control conditions, participants simply visited all 25 patches, leaving each when they chose to under the instruction to collect as many good berries and as few bad berries as possible. Thus, each patch was foraged once. There was no time limit for the Control condition.

On the second visit in the Choice and Forced Move conditions, the previously visited patches were presented again, sequentially. Participants picked among the remaining berries and left each patch when they wanted to move to the next. The second visit in the field ended once all the patches from the first phase were viewed again.

### Results and discussion

Before analysis, 1.83% of the clicks were excluded because the RT was too short (less than 200 ms) or too long (longer than 4 s). As the experiment design required participants to visit all the patches twice, we did not need to exclude any patches that were not visited twice, unlike Experiment 2. As in Experiments 1 and 2, we used non-parametric statistical tests for some analyses that did not satisfy the normality assumption. The descriptive information of Experiment 3 is depicted in Table 5.

**Table 5:** Mean (standard deviation) descriptive information of Experiment 3

Moving type	*N*	Patches visited		Clicks per patch		Average hit rate		Average FA rate	
		First/total	Second	First/total	Second	First/total	Second	First/total	Second
Forced Move	20	25.0 (0.00)	23.9 (3.39)	14 (4.93)	17.25 (7.82)	0.27 (0.09)	0.31 (0.13)	0.01 (0.01)	0.02 (0.02)
Choice Move	20	23.5 (1.82)	23.6 (1.67)	12.28 (5.08)	13.27 (8.02)	0.24 (0.10)	0.25 (0.14)	0.01 (0.01)	0.02 (0.02)
Control	20	25.0 (0.00)		33.79 (11.65)		0.62 (0.20)		0.06 (0.05)	

“First” and “Second” refer to the foraging periods before and after interruption. “Total” refers to the foraging period of Control condition. Hit = proportion of picked berries that were “good”. FA = proportion of picked berries that were “bad”

**Fig. 8:**
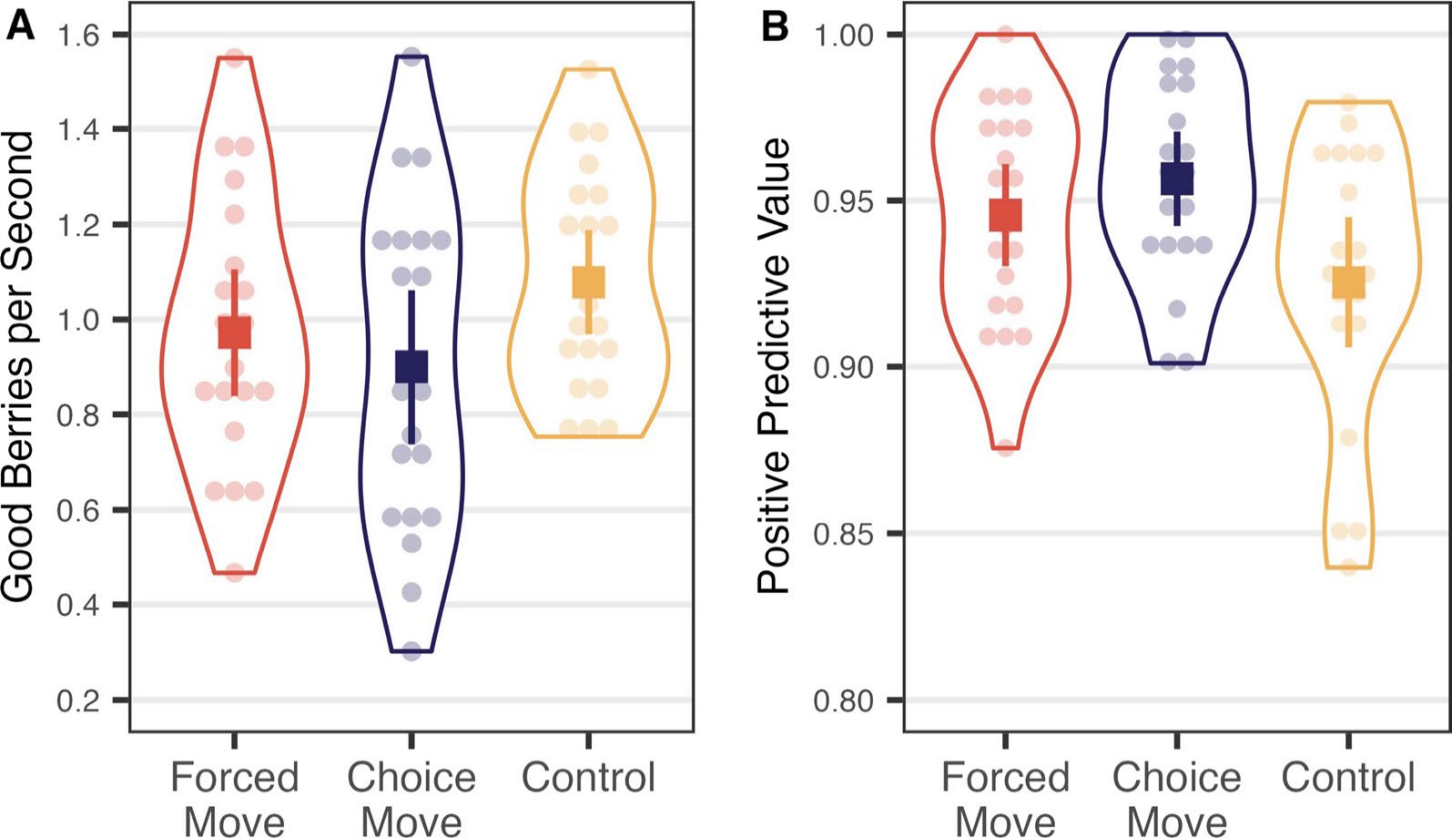
**A** Overall rate of return and **B** PPV of Experiment 3: *Note*. The graphs contain individual data points (transparent circles) and summarized data points (solid squares). Error bars are 95% confidence intervals

Figure 8 shows the average rate of return and the PPV for each participant in the three conditions. The overall rate of return, *F*(2, 57) = 1.875, *p* = 0.163, *η*^2^_*p*_ = 0.062, and PPV, *χ*^2^(2) = 5.816, *p* = 0.055, *ε*^2^ = 0.099, did not show significant differences across the conditions, though the PPV analysis just misses the 0.05 level with a trend toward lower PPV in the Control condition.

**Fig. 9:**
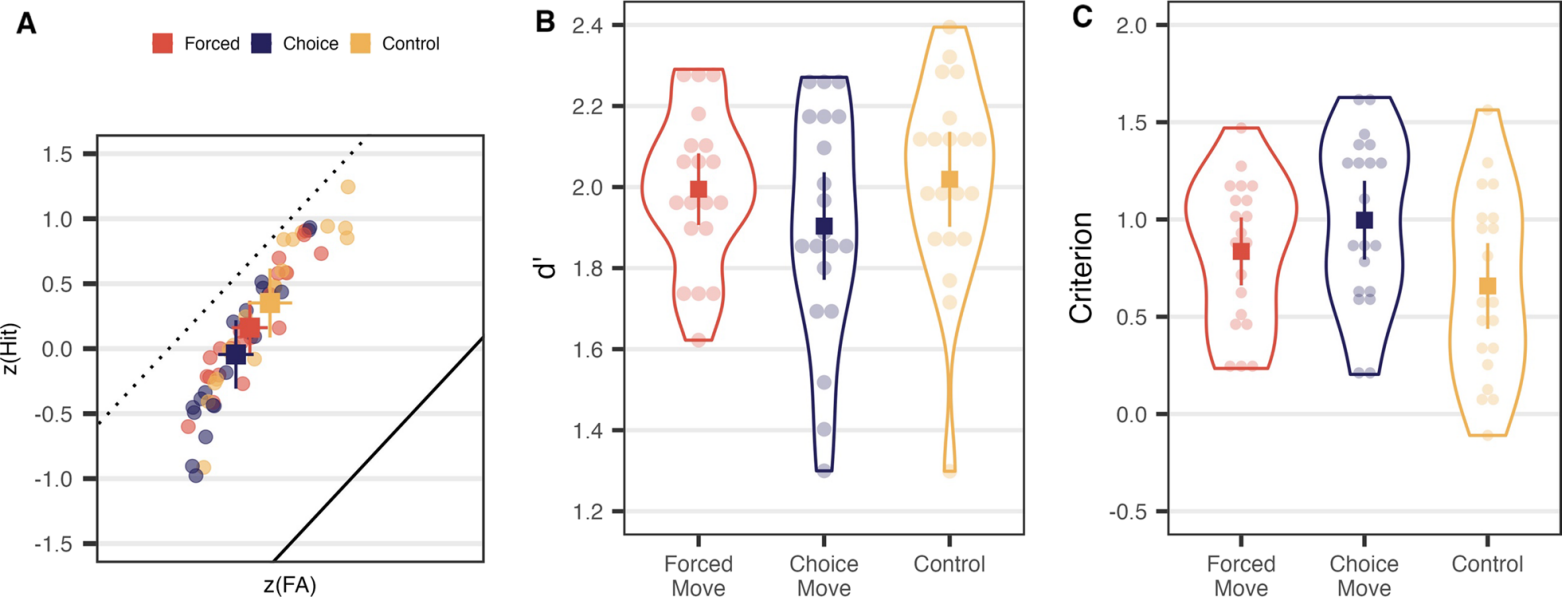
Signal detection analysis in Experiment 3: **A** zROC. Transparent dots are individual points, and solid squares are summarized points. Error bars are 95% confidence intervals. The solid diagonal line indicates expected performance when *d*′ is 0 (chance). The dotted diagonal line indicates expected performance when *d*′ is 2.5. **B** *d*′ of each condition. **C** Response criterion of each condition

Figure 9 shows *d*′ and criterion values for each participant in each condition. *d*′ did not vary significantly different across conditions, *χ*^2^(2) = 2.361, *p* = 0.307, *ε*^2^ = 0.040. Criterion showed a marginal effect with the Control condition being most liberal and the Choice Move condition being most conservative, *F*(2, 57) = 3.136, *p* = 0.051, *η*^2^_*p*_ = 0.099.

**Fig. 10:**
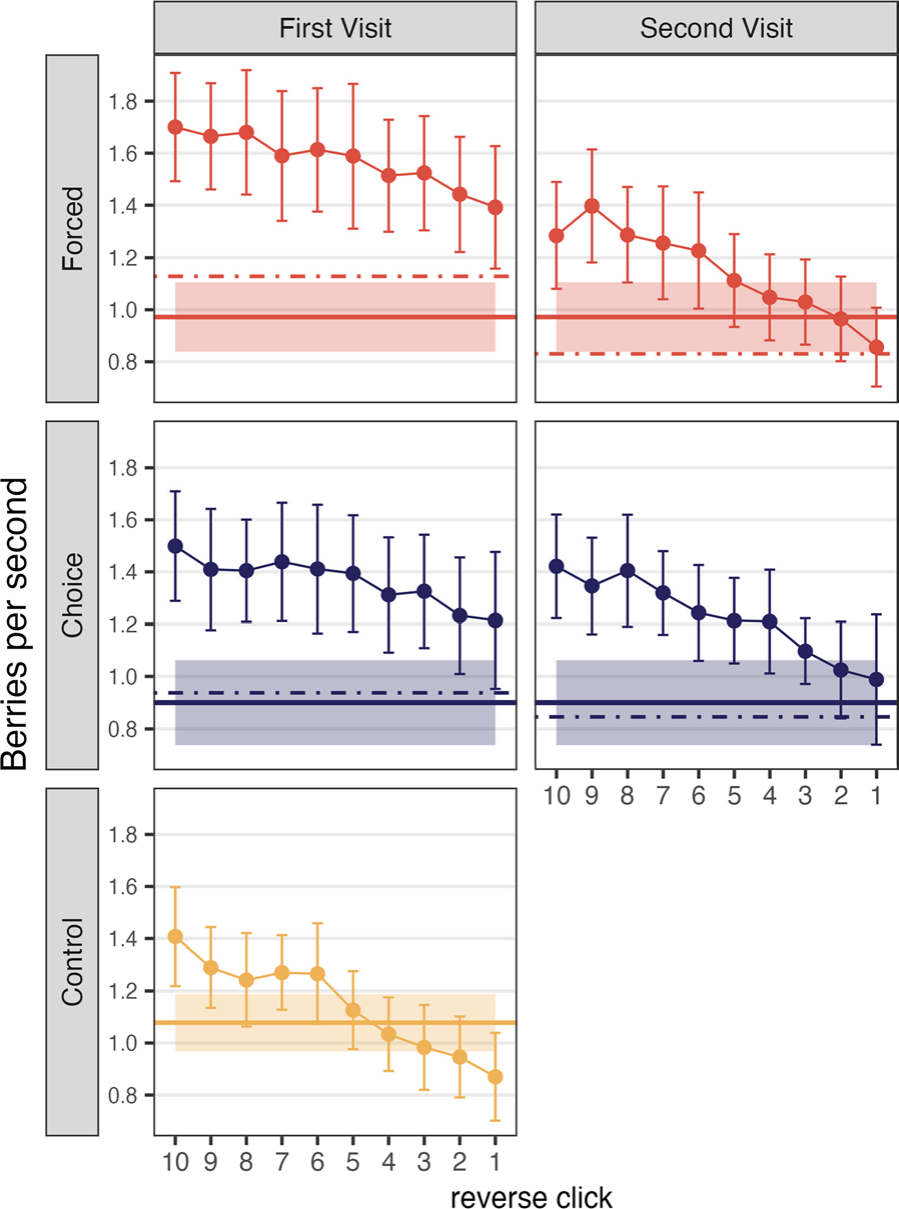
Instantaneous rate of return, or expected number of berries per second, as a function of reverse click order in Experiment 3: Solid dots in the graphs for each condition indicate the average rate of return for each click. Error bars depict 95% confidence intervals. Solid horizontal lines are the average rates of return from the entire habitat, and shaded areas are 95% confidence intervals. Dashed lines are the average rate of return from each visit

Figure 10 shows the instantaneous rate for the last 10 clicks in both the first and second portions of the Forced and Choice conditions. As in Experiment 2, the Control condition shows some overharvesting. The first sections of the Forced and Choice conditions show “underharvesting” because participants were encouraged or forced to leave each patch before it was fully depleted. When participants returned to those patches, they continued to pick, leaving the patches in an MVT-optimal manner when the instantaneous rate dropped to or just below the overall rate (Table 6).

**Table 6:** Total rate and instantaneous rates of return from each reverse click order in Experiment 3

Moving type	Total rate (SD)	Click 5	Click 4	Click 3	Click 2	Click 1
Forced Move	0.97 (0.29)	1.11*	1.05	1.03	0.96	0.86
Choice Move	0.90 (0.35)	1.21***	1.21***	1.10**	1.02	0.99
Control	1.08 (0.24)	1.13	1.03	0.98	0.95	0.87*

^*^p < .05, **p < .01, ***p < .001. *p* values were adjusted with the Bonferroni method

## General discussion

Across three experiments, we found that foraging was not markedly disrupted by interruption. The interrupted conditions produced similar rates of return, similar PPVs, and similar patch-leaving strategies to those produced by the uninterrupted Control conditions. Foraging behavior was resistant to interruption whether the target collection resumed immediately after interruption (Experiment 1) or later after many patches had been visited (Experiment 3). In Experiments 2 and 3, participants in general stayed somewhat longer in a patch than what would be predicted by MVT. This overharvesting is often found in animal and in human foraging (Carter & Redish, 2016; Le Heron et al., 2020).

The Choice Move condition of Experiment 2 was the one case where behavior after interruption appeared to be changed. Here the second visit produced a lower rate of return, poorer collection quality, and apparently suboptimal patch-leaving. This might be the result of the task demands, in which participants were allowed to exhaust patches in the first phase and then, unexpectedly, told to revisit the depleted patches. Alternatively, participants might have based their estimate of the post-interruption overall rate on the rate from only the depleted patches. These two accounts are not mutually exclusive. In any case, when the task rules of the Choice Move condition from Experiment 3 were changed to rush participants to through the patches on their first visit, participants left more targets behind on their first visit. With more berries worth picking on the second visit, the Experiment 3 Choice Move condition produced results comparable to the other conditions.

Despite interruptions, our results reveal resilience in foraging behavior. What mechanisms support this stability? One might speculate that some extended spatial memory (Chun & Jiang, 1998; Geng & Behrmann, 2005) might play a role, allowing participants to continue target collection from where they left off before the interruption, as proximity is highly predictive of which target item will be selected next (Clarke et al., 2022a, 2022b). However, this is unlikely considering that visual search relies more on current perceptual processing rather than on memory for rejected distractors or bad berries (Horowitz & Wolfe, 1998). It is more likely that this resilience reflects a solid ability to estimate the overall rate of return. Our observations are consistent with a broader body of research indicating that the quitting rule in foraging is oriented toward the goal of outcome maximization, even when foraging does not occur in a spatial layout (e.g., Hutchinson et al., 2008; Kane et al., 2022; Wilke et al., 2009). Such findings contribute to a growing understanding of the adaptability of human foraging behavior, suggesting that even when faced with disruptions, humans are capable of adjusting their strategies to continue foraging efficiently.

Previous studies have reported clearly negative consequences of interruption in other types of visual search tasks. Disruptive interruption can arise from abrupt onsets (Yantis & Jonides, 1984), concurrent perceptual (Shen & Jiang, 2006) or working memory tasks (Alonso et al., 2021). Moreover, interruption can be costly in real-life situations. It can delay visual search in medical imaging (Drew et al., 2018; Williams & Drew, 2017) and has been implicated as a factor in more than 8% of deaths while driving (Stewart, 2021). In contrast, this study showed that visual foraging, unlike other search tasks, was resistant to interruption; at least, to the interruptions that were used here. While continuous and spatial interruptions affect working memory capacity and target template switching in visual foraging (Thornton et al., 2021), temporal pauses do not significantly impact patch-leaving decision or overall outcomes of collection.

Previous studies have demonstrated the adaptability of visual foraging in a complex world. Despite huge individual differences in foraging patterns (Clarke et al., 2022a, 2022b; Irons & Leber, 2018), foragers adapt their behavior to the changing environment by favoring valuable targets (Wolfe et al., 2018), synchronizing foraging duration with patch quality (Fougnie et al., 2015; Zhang et al., 2017), flexibly adjusting visual working memory capacity by task demands (Kristjánsson et al., 2018), and considering the potential expectation of instantaneous and total gains (Ehinger & Wolfe, 2016). In these various conditions, foragers are still able to roughly optimize patch-leaving despite fluctuating circumstances. The present study provides additional support of the adaptability of visual foraging behavior. Foragers can continue to follow MVT-style or analogous patch-leaving predictions even when faced with interruptions of foraging.

The ubiquity of roughly MVT-optimal foraging behavior across situations and species suggests a behavior shaped by evolutionary processes. Roughly optimal foraging is widely reported in other animal species (Bond, 1983; Ranc et al., 2021; Shochat et al., 2004) and across human development (Gil-Gómez de Liaño et al., 2022; Wiegand et al., 2019). Foraging behavior strikes a balance between energy intake and energy use, allowing foragers to maximize net gain, at least in relatively simple tasks like berry picking (perhaps, especially, in on-line berry picking in a very regular field). Forces in the real world will complicate foraging situations. Berry picking behavior will change if there are other pickers in the field or if there is a wolf, engaging in own “foraging” behavior (Thornton et al., 2021). In human foraging tasks, behavior will be quite different if the task puts high value on finding all targets, rather than maximizing the rate (Jóhannesson et al., 2016; Kristjánsson et al., 2020). In the radiology reading room, for example, radiologists may be encouraged to stay longer on a case than MVT might predict if their task is to find all the metastases. This is weakly mirrored in the Choice condition of Experiment 2 where participants stayed in the patch longer than MVT would predict because they were responding to different task demands. The extent of this behavioral flexibility would be worth further study.

In summary, human visual foraging performance appears to be strongly influenced by a deeply ingrained set of rules that foragers adhere to, even when foraging is interrupted. These rules may or may not resemble those of MVT—importantly, foragers achieved similar outcomes by following similar patch-leaving rules. Future research could examine the effect of spontaneous interruptions, or a self-initiated pause, as opposed to externally imposed ones, on foraging behavior. In addition, the cognitive processes or external circumstances that lead to deviations from MVT should be addressed.

## Data Availability

The datasets generated and analyzed during the current study are available in the OSF repository, https://osf.io/prne8/.

## Abbreviations

MVT: Marginal value theorem
RT: Reaction time
